# Immunosuppressive treatment patterns in kidney transplant recipients in France: an insurance claims database study (OISTER) over a 12 year period

**DOI:** 10.1007/s40620-025-02296-4

**Published:** 2025-05-13

**Authors:** Dany Anglicheau, Antoine Durrbach, Isabelle Bardoulat, Mickael Arnaud, François-Emery Cotté, Radu Vadanici, Mélanie Chartier, Denis Glotz

**Affiliations:** 1 Service de Transplantation Rénale, Hôpital Necker, AP-HP, Paris, France; 2 Faculté de Médecine, Université de Paris Cité, Paris, France; 3 INSERM U1186, Institut Gustave-Roussy, Université Paris-Saclay, Villejuif, France; 4 Service de Néphrologie et Transplantation, Hôpital Henri Mondor, AP-HP, Creteil, France; 5 IQVIA, Paris, France; 6 IQVIA, Bordeaux, France; 7 Bristol-Myers Squibb, 3 rue Joseph Monier, 92500 Rueil-Malmaison, France; 8 Paris Translational Research Center for Organ Transplantation, INSERM UMR 970, Paris, France; 9 Service de Néphrologie et Transplantation, Hôpital Saint-Louis, AP-HP, INSERM U1160, Paris, France

**Keywords:** Kidney transplant, Immunosuppressive treatment, Calcineurin inhibitor, Belatacept

## Abstract

**Background:**

The impact of new immunosuppressive strategies to prevent graft rejection on prescribing patterns is poorly documented. The objective of this study was to describe immunosuppressive medication use and survival outcomes in patients undergoing kidney transplantation in France between 2009 and 2020.

**Methods:**

This retrospective cohort study was performed using data from the French national healthcare database. All adult patients undergoing kidney transplantation between 2009 and 2020 were identified, and followed from transplantation until study end, death or graft failure. All immunosuppressive drug deliveries from pharmacies were documented. Survival and death-censored graft failure were estimated using Kaplan-Meier analysis.

**Results:**

Thirty four thousand six hundred kidney transplantation patients were eligible. Median follow-up duration was 4.0 years [IQR: 1.6–7.0 years]. Five-year survival probability was 0.890 [0.885–0.895], and death-censored graft survival was 0.850 [0.844–0.856]. Overall survival, but not graft survival was age-dependent. Calcineurin inhibitors were delivered to 29,573 patients (91.3%), antimetabolites to 29,318 (90.5%), corticosteroids to 28,536 (88.1%), mTOR inhibitors to 5231 (16.1%) and belatacept to 1272 (3.9%). The use of tacrolimus, everolimus and belatacept increased over time, while the use of corticosteroids, ciclosporin and sirolimus declined. Immunosuppressive treatment was maintained by 22,963 patients (76.5%) throughout follow-up.

**Conclusions:**

In this comprehensive study of kidney transplant recipients in France, we observed high rates of overall patient and graft survival. Over the study period, immunosuppressive treatment patterns were relatively stable and dominated by the use of calcineurin inhibitors. These results provide important insights into the state of post-transplant care and the potential implications of increased availability of innovative therapies.

**Supplementary Information:**

The online version contains supplementary material available at 10.1007/s40620-025-02296-4.

## Introduction

Kidney transplantation is the recommended and most cost-effective way to manage kidney failure [1, 2]. Patients undergoing kidney transplantation require lifelong maintenance immunosuppressive therapy, starting immediately after the transplant, in order to prevent graft rejection [2].

The current treatment paradigm combines different classes of immunosuppressive drugs with complementary pharmacological targets to provide an optimal immunosuppressive therapy [3]. In 2022, the European Association of Urology (EAU) published guidelines which recommend initiating maintenance treatment with a calcineurin inhibitor (CNI), an antimetabolite and corticosteroids [2]. In the event of nephrotoxicity or other related adverse effects of CNIs, which are common and often dose-dependent [3], a switch to an alternative immunosuppressive treatment may be necessary [2].

Belatacept is a fusion protein of the extracellular domain of the immune checkpoint protein cytotoxic T-lymphocyte-associated protein 4 (CTLA-4) and constant domains of immunoglobulin [4]. Belatacept has been approved in the European Union for the prevention of graft rejection following kidney transplantation since 2011. The benefit-risk profile of belatacept in the kidney transplantation setting appears to be similar to that of tacrolimus in the de novo setting [5–7] although the two therapeutic strategies have not been compared directly in large randomized clinical trials [6]. In the conversion setting, several studies have shown that switching from a CNI to belatacept is associated with lower graft failure rates or improved kidney function compared to continuation of the initial CNI treatment [8–12]. Compared to ciclosporin, immunosuppressive therapy with belatacept is associated with a lower incidence of hypertension, hyperlipidemia and new-onset diabetes after transplantation [5], as well as with a low risk of opportunistic infections, which occur principally in patients switched to belatacept in the first six months following kidney transplantation [13].

As immunosuppressive therapies reduce general immune responses, viral replication remains an important issue in solid organ transplantation. Since 2018, clinical trials integrating mTOR inhibitors (mTORi) in association with CNI or belatacept have suggested a lower level of replication of cytomegalovirus or BK polyoma virus with these regimens [14]. For this reason, mTORi have become integrated in immunosuppressive treatment panels. In addition, new treatment options have become available for patients requiring immunosuppression depending on the state of the graft. Current practice is to personalize immunosuppressive treatment with the goal of limiting specific adverse effects by taking into account the age and comorbidities of the patients. Although it is likely that physicians have changed their prescribing practice over the last decade, this remains to be documented. Currently, available data on how immunosuppressive drugs are prescribed following kidney transplantation in the real-world setting are limited, and no such data are available for France. In the present study, we have investigated immunosuppressive prescription patterns since 2009 using data from the French National Health Data System, the *Système National des Données de Santé* (SNDS). The objective of this study (OISTER) was to describe real-world patterns of immunosuppressive use and survival outcomes in patients who underwent kidney transplantation between 2009 and 2020 in France with a focus on belatacept.

## Methods

### Study design

The OISTER study is a retrospective cohort study performed using data from the SNDS database. Data were extracted for all adult patients who underwent kidney transplantation between 1^st^ January, 2009 and 31^st^ December, 2020. The index date for each patient was the date of first kidney transplantation during the study period. Participants were followed from the index date until death, graft loss, or end of the study period, whichever occurred first. A historical period prior to the index period going back to 1^st^ January, 2008 was collected to retrieve information on relevant medical comorbidities.

### Data sources

The SNDS is the French National Health Data System (national health insurance claims database), which currently covers >99% of the French population [15, 16]. Healthcare consumption can be followed across each beneficiary’s lifetime using a unique patient identifier. The SNDS contains pseudo-anonymized information on all hospital and community healthcare consumption in the public and private sectors reimbursed by public national health insurance.

### Study participants

The study included all adults hospitalized for kidney transplantation (CCAM procedure codes JAE003 or HNEA002) during the study period (2009–2020). Exclusion criteria were age <18 years, no gender documented, absence from the SNDS database during the year prior to the index date, and data inconsistencies.

In order to evaluate the quality of coding of these procedures in the SNDS, the number of transplantations performed each year was compared to the number of transplantations documented by the *Agence de la Biomédecine*, the agency responsible for coordinating organ transplantation in France [17].

### Study variables

Demographic variables extracted from the database were limited to date of birth, date of death (if the patient died) and gender. Absence of immunosuppressive treatment for more than three months, new transplantation or resumption of dialysis sessions for at least three months was used as a proxy variable for graft loss. Selected cardiovascular and metabolic comorbidities were assessed in the year preceding the date of kidney transplantation using diagnostic codes based on the International Classification of Diseases, 10th Revision (ICD-10 codes) classification in hospital discharge summaries or providing access to long-term disease status, Anatomical Therapeutic Chemical (ATC) classification codes for prescribed drugs and French medical procedure codes, as listed in Online resource Table S1.

Immunosuppressive treatments delivered in community retail pharmacies (CNIs, antimetabolites, mTOR inhibitors and corticosteroids) were identified in reimbursement claims using the relevant ATC and Presentation Identifier codes. Since belatacept is delivered only by hospital pharmacies, it could not generally be identified by name. However, delivery by the retrocession procedure was approved in July 2020 and from this date forward, it was possible to identify belatacept directly in the SNDS database. Before this date, patients treated with belatacept were identified using an algorithm constructed on the basis of a feasibility study [18]. This required patients to have been hospitalized at least three times within a 90-day period for delivery of chemotherapy for a non-tumoral disease with an associated or related diagnostic code for kidney transplantation, and at least one delivery of an mTOR inhibitor or an antimetabolite within 90 days of the index hospitalization and no delivery of a CNI during the fourth month following the index hospitalization. The algorithm was also used after July 2020 to identify patients administered belatacept in hospital. In case of a switch between treatments, the initial treatment was considered to have been stopped on the date of first delivery of a relay treatment. Treatments were considered as being used in combination when more than one treatment was delivered for a duration of ≥30 days and if the combination was a recognized immunosuppresive treatment regimen.

Event-free survival was defined as the interval between the index date and the date of all-cause death, graft loss, loss to follow-up or exit from the risk pool. Graft survival was defined as the interval between the index date and the date of new transplantation.

### Statistical analysis

Continuous variables are described by mean values with their standard deviation (SD) or median values with their interquartile range (IQR). Categorical variables are presented as frequency counts and percentages, with their 95% confidence intervals (CI). Time-to-event survival analysis was performed using a Kaplan-Meier function for overall survival and a cumulative incidence function estimate for graft survival, taking into account death as a competing risk. All analyses were performed using SAS^®^ software version 9.4 (Cary, USA).

## Results

### Patients

**Fig. 1:**
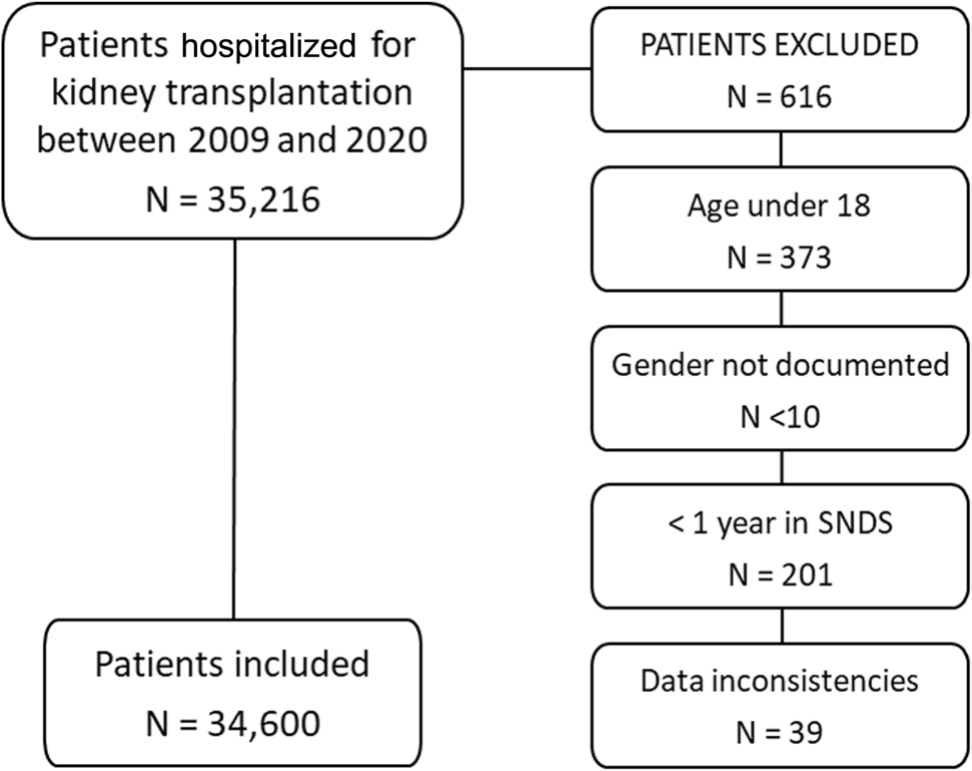
Patient flow

Between 2009 and 2020, 35,216 patients were hospitalized for kidney transplantation, of whom 34,600 were eligible for the study. A patient flow diagram is provided in Figure 1. The main reasons for exclusion were age <18 years (60.5%) and <1 year of follow-up in the SNDS (32.6%). The number of transplant procedures per year gradually increased starting in 2009, and then fell in 2020 during the COVID pandemic (Online resource Figure S1). On a year-to-year basis, the number of identified patients closely matches the number of kidney transplantations in the national transplantation registry maintained by *Agence de la Biomédecine* (Online resource Figure S1). Over the study period, the median follow-up of transplant recipients was 4.0 years [IQR: 1.6–7.0 years].

The characteristics of the patients at the index date are presented in Table 1. These are restricted to patients with an index date between 2009 and 2019, since data for 2020 may not be representative due to delayed or incomplete reporting during the COVID pandemic. The majority of transplant recipients were men (62.7%) and their mean age was 53.0 ± 14.2 years. Cardiovascular and metabolic comorbidities were frequent, especially hypertension (92%) and dyslipidemia (46%) (Table 1). Overall, 1829 patients (5.3%) had already received a kidney transplant prior to the index date and 1149 patients (3.5%) underwent kidney transplantation as part of a double organ transplant. Patients who had received belatacept were very similar to the overall cohort in terms of age, gender, comorbidities and transplantation history.

**Table 1:** Patient characteristics at the index date (population 2009-2019)

	All patients	Belatacept
	N = 32,391	N =1272
Gender		
Male	20,305 (62.7%)	740 (58.2%)
Female	12,086 (37.3%)	532 (41.8%)
Age (years)		
Mean ± SD	53.0 ± 14.2	52.3 ± 14.9
By class (%)		
18-29	2190 (6.8%)	108 (8.5%)
30-39	4016 (12.4%)	174 (13.7%)
40-49	6190 (19.1%)	240 (18.9%)
50-59	8075 (24.9%)	270 (21.2%)
60-69	7992 (24.7%)	319 (25.1%)
≥70	3928 (12.1%)	161 (12.7%)
Comorbidities^b^		
Hypertension	29,756 (91.9%)	1171 (92.1%)
Dyslipidemia	14,947 (46.1%)	590 (46.4%)
Diabetes	6339 (19.6%)	215 (17.3%)
Ischemic heart disease	5222 (16.1%)	198 (15.6%)
History of obesity or bariatric surgery^c^	3719 (11.5%)	153 (12.0%)
Heart failure	2918 (9.0%)	140 (11.0%)
Peripheral artery disease	2301 (7.1%)	68 (5.3%)
Malnutrition	2200 (6.8%)	83 (6.5%)
History of stroke	615 (1.9%)	34 (2.7%)
Transplantation history		
Multiple transplantation (kidney + other organ)	1149 (3.5%)	24 (1.9%)
Prior kidney transplantation	1735 (5.4%)	69 (5.4%)
Prior non-kidney organ transplantation	2308 (7.1%)	73 (5.7%)
Pre-emptive transplantation	4286 (13.2%)	149 (11.7%)

^a^The description of patient management refers to the period between 2005 and 2019 (2020 being considered unrepresentative due to the COVID pandemic). Nevertheless, comparison of the patient populations transplanted in 2005-2020 and in 2005-2019 showed them to be similar

^b^Comorbidities were identified as described in the Methods section for the year preceding the date of kidney transplantation (index date)

^c^Patients with a history of obesity (ICD-10 code: E66) or with a history of bariatric surgery (procedure code)

### Outcome

Patient and graft survival at five years was high, with most patients being alive with a functioning graft at study end. At five years, overall survival was 89.0% and death-censored graft survival was 85.0% (Table 2). Overall survival decreased with age from 97.8% for the youngest age group (18–29 years) to 73.1% for the oldest (≥70 years) (Table 2). Graft survival was similar in all recipient age groups (84.2% for the youngest age group and 80.6% for the oldest). Kaplan-Meier survival curves for overall survival and graft survival are provided in Online resource Figure S2.

**Table 2:** Clinical outcomes

Age (class)	Number of patients	Event-free survival probability^b^ at five years (95%CI)	Graft survival probability^c^ at five years (95%CI)
All	16,139^a^	0.890 [0.885-0.895]	0.850 [0.844-0.856]
18-29	1177	0.978 (0.968-0.985)	0.842 (0.819-0.862)
30-39	2010	0.971 (0.963-0.978)	0.866 (0.850-0.880)
40-49	3143	0.952 (0.944-0.959)	0.866 (0.854-0.878)
50-59	4178	0.903 (0.894-0.912)	0.873 (0.862-0.883)
60-69	4095	0.823 (0.811-0.834)	0.823 (0.811-0.835)
≥ 70	1,536	0.731 (0.708-0.752)	0.806 (0.784-0.826)

^a^In order to ensure five full years of follow-up, only patients with an index date prior to 1^st^ January, 2015 have been evaluated

^b^Determined with a Kaplan-Meier survival function

^c^Determined with a cumulative incidence function to take into account the competing risk of death

*CI* confidence interval

### Immunosuppressive treatments

Overall, it was possible to document the prescription of at least one immunosuppressive drug over the follow-up period (2009–2019) in 29,933 transplanted patients (92.4%) (Table 3). At least one CNI was used by 29,573 patients (91.3% of transplanted patients; 98.8% of patients with a documented treatment), principally tacrolimus. The second most frequently used immunosuppressive class was antimetabolites, almost exclusively mycophenolic acid, mainly used in combination with a CNI. In 16.1% of cases, mTOR inhibitors (principally everolimus) were used and belatacept was used in 3.9%. Corticosteroids were used by 88.1% of transplanted patients.

**Table 3:** Immunosuppressive treatment during the follow-up period

Treatment	Year of index kidney transplantation			
	Overall (*N* = 32,391)	2009–2011 (*N* = 7760)	2012–2015 (*N* = 11,518)	2016–2019 (*N* = 13,113)
At least one IS drug	29,933 (92.4%)	7161 (92.3%)	10,701 (92.9%)	12,071 (92.1%)
CNI	29,573 (91.3%)	7082 (91.3%)	10,564 (91.7%)	11,927 (91.0%)
Tacrolimus	26,051 (80.4%)	5874 (75.7%)	9239 (80.2%)	10,938 (83.4%)
Ciclosporin A	6518 (20.1%)	2110 (27.2%)	2587 (22.5%)	1821 (13.9%)
Corticosteroids	28,536 (88.1%)	6898 (88.9%)	10,245 (88.9%)	11,393 (86.9%)
Anti-metabolites	29,318 (90.5 %)	7098 (91.5%)	10,509 (91.2%)	11,711 (86.3%)
Mycophenolic acid	28,900 (89.2%)	7029 (90.6%)	10,341 (89.8%)	11,530 (87.9%)
mTOR inhibitors	5231 (16.1%)	1560 (20.1%)	1859 (16.1%)	1812 (13.8%)
Belatacept	1272 (3.9%)	172 (2.2%)	587 (5.1%)	513 (3.9%)
Combination therapy				
CNI + antimetabolite	28,837 (89.0%)	7001 (90.2%)	10,338 (89.8%)	11,498 (87.7%)
CNI + mTOR inhibitor	3598 (11.1%)	678 (8.7%)	1322 (11.5%)	1598 (12.2%)
mTOR inhibitor + antimetabolite	1789 (5.5%)	1004 (12.9%)	586 (5.1%)	199 (1.5%)
CNI + mTOR inhibitor + antimetabolite	846 (2.6%)	393 (5.1%)	287 (2.5%)	166 (1.3%)
Belatacept + antimetabolite + CS	985 (3.0%)	125 (1.6%)	462 (4.0%)	398 (3.0%)

Percentages for the individual treatments are calculated with respect to all transplanted patients during each period

*IS* immunosuppressive, *CNI* calcineurin inhibitor, *CS* corticosteroid, *mTOR* mammalian target of rapamycin

We also analyzed treatment patterns according to the year during which the kidney graft was performed. The extent of prescribing for each treatment by calendar year is presented in Online resource Figure S3. We observed some shift in prescribing from ciclosporin (reduced from 22.0% of patients in 2009 to 14.3% in 2019) to tacrolimus (increased from 66.2% in 2009 to 79.2% in 2019), and an increase in the proportion of patients delivered a CNI in combination with an mTOR inhibitor (from 1.1% to 7.1%, respectively). Over the decade, everolimus replaced sirolimus as the main mTOR inhibitor (increasing from 27.0% of mTOR inhibitor prescription in 2009 to 87.7% in 2019), accounting for >90% of prescriptions in this period. Belatacept was introduced in 2011 and 2.7% of patients were receiving it in 2018; belatacept accounted for 14.6% of all switches between 2012 (the first full year of belatacept availability) and 2015 to 17.1% between 2016 and 2020.

The majority of patients remained on their initial immunosuppressive treatment throughout the follow-up period. The remaining 6970 (23.5%) patients converted to a relay treatment at some stage and 2371 did so more than once (8.0%). The most frequent first relay treatments were mTOR inhibitor-based regimens (*N* = 3111), followed by ciclosporin-based regimens (*N* = 1423), tacrolimus-based regimens (*N* = 1389) and belatacept-based regimens (*N* = 887). Switching patterns are listed in Online resource Table S2. Apart from switching, the most frequent treatment adaptions were add-on of an mTOR inhibitor to tacrolimus (*N* = 1552; 6.5% of patients commencing treatment with tacrolimus) or to ciclosporin (*N* = 397; 8.5% of patients commencing treatment with ciclosporin).

## Discussion

This study evaluated patterns of immunosuppressive treatment in all kidney transplant recipients in France included in the SNDS between 2009 and 2020. The observed therapeutic strategies are aligned with French and international recommendations, with a CNI accounting for >95% of initial treatments, in the majority of cases in association with an antimetabolite and a corticosteroid. In addition, maintenance therapy continued unchanged throughout the treatment period in over three-quarters of the patients. Immunosuppressive treatment regimens remained generally stable between 2009 and 2019.

The main strength of the study is the completeness of capture in the SNDS of patients undergoing kidney transplantation in France. The 34,600 identified patients closely match the 38,733 patients registered in the *Agence de la Biomédecine* national transplantation registry. The two sources differ by <5%, which can be considered to be within an acceptable margin of error. Another strength of the study is that the use of maintenance immunosuppressive treatments can be followed over a ten-year period since the SNDS includes exhaustive reimbursement data for individual patients. Over the study period, the number of kidney transplantations performed each year increased, with more than 3200 transplants being carried out annually between 2016 and 2019. These numbers are consistent with those reported by the *Agence de la Biomédecine* (Online resource Figure S1). However, during the COVID-19 pandemic in 2020, the number of transplantations fell to pre-2009 levels. Such a reduction in the number of transplantations performed during this period has also been reported in many other countries [19].

We observed that overall survival was 89% at five years and, as expected, declined with the age of the graft recipient. In addition, graft survival was high, 85% at five years. This finding is consistent with other studies in the international literature, with survival being slightly higher than that reported in a recent meta-analysis including 86 studies performed between 1999 and 2020, which provided five-year estimates of 81.2% [95% CI 78.7%–83.6%] for overall survival and 80.0% [77.6%–82.4%] for graft survival, despite the increasing age of donors and the frequent comorbidities [20]. In our study, graft survival was comparable irrespective of the age of the graft recipient.

We documented immunosuppressive treatment in 92.4% of all grafted patients. In principle, all kidney transplant recipients should receive lifetime immunosuppressive treatment. The main reason for which treatment of a small minority of several thousands of patients was not identified may be related to how treatments are documented in the SNDS database; another reason might be due to the inclusion of patients in clinical trials in which immunosuppressives were directly distributed to the patient. Apart from the most expensive treatments, individual treatments delivered in hospital cannot be identified. Only medications delivered in community pharmacies are documented by the name of the drug itself. For this reason, if patients died in hospital or lost their grafts early on before returning home and before they had the opportunity to be delivered immunosuppressive therapy from a community pharmacy, then any immunosuppressive treatment delivered in hospital would not be identified. This explanation seems likely, since, in a previous cross-sectional study of the SNDS performed in 2013, in which patients who died during the transplantation hospital stay were excluded, 99.4% of recipients had been delivered an immunosuppressive drug at least once in the year following transplantation [21].

As recommended in the EAU practice guidelines [2], the initial treatment strategy was a combination of a CNI with an antimetabolite in >90% of patients, whereas mTOR inhibitors and belatacept were mainly used as a relay treatment after a switch from the initial immunosuppressive treatment. Tacrolimus-based regimens were the most frequently prescribed initial treatments (88.1%), those for which persistence throughout the follow-up period was highest (80.5%), and the most frequent relay treatment for patients switching from another initial immunosupressive regimen (68.3%). The low level of belatacept use observed (<5% of patients) is consistent with what was previously observed in the United States [22] and in a recent French study on the management of kidney transplant recipients infected with COVID-19 in 2020 [23].

Over the decade, little change in immunosuppressive treatment patterns was observed. Prescription of tacrolimus increased at the expense of ciclosporin, and everolimus had practically replaced sirolimus by the second half of the decade [24]. The use of a CNI in association with an mTOR inhibitor increased but this combination never exceeded 10% of patients. The proportion of patients receiving mTOR inhibitors or belatacept also increased, principally following a switch from the initial immunosuppression. Although one quarter of patients were switched at some stage during the study period, less than 10% required switching a second time. The similarities in patterns of immunosuppression prescription between the OISTER study integrating more recent years (up to 2019), and earlier French cross-sectional studies, dating from 2013 and 2014 [21, 25], underline the stability of immunosuppressive treatment patterns over the last decade.

Changes to the availability of belatacept in France in 2020, through the retrocession procedure whereby the patient can be treated without having to go to hospital, may make belatacept more attractive to prescribers and patients and lead to an increase in use. In addition, considerable cost savings may be expected from moving treatment outside the hospital, which may also influence prescription. However it is too early to evaluate the consequences of this change on prescription rates for belatacept. Unlike a previous retrospective international study [9], we did not find that patients who switched to belatacept remained on treatment until the end of follow-up more frequently than those who switched to an mTOR inhibitor.

The main limitations of the study are inherent to the data source. For example, no information is available on why patients initiated, switched or discontinued immunosuppressive treatment, and this limits interpretation of the treatment patterns. For example, it is not possible to know whether treatment changes were triggered by safety issues, or whether the choice of treatment was influenced by potential prognostic factors such as viral status, graft origin, kidney function or HLA compatibility. Another limitation relates to the fact that the SNDS contains no information prior to 2008. For this reason, relevant events, such as previous kidney transplants, are likely to be underestimated in case of multiple transplantations. Patients receiving a second kidney transplant may well be treated more intensively than those undergoing their first graft transplant. A third limitation is that it was not possible to identify patients receiving belatacept directly for most of the study duration since this treatment was administered in hospitals, where individual medications are not documented in the SNDS. Therefore, we used an algorithm-based proxy to capture belatacept use. This algorithm was built from expert consensus, but could not be independently validated. Overall survival may have been somewhat underestimated, as death was not documented exhaustively in the SNDS at the beginning of the treatment period.

In conclusion, the OISTER study provides a broad picture of immunosuppressive treatment strategies used for kidney transplant maintenance nationwide over the previous decade. During this period, treatment patterns remained relatively stable, and were dominated by the use of CNIs. Since access to belatacept was facilitated in 2020 through the retrocession program, it will be of interest to document whether use of this immunosuppressive drug increases as a result of this change over the coming decade, and whether this will have an impact on clinical outcomes.

## Supplementary Information

Below is the link to the electronic supplementary material.

Supplementary file1 (DOCX 364 KB)

## Data Availability

Access to SNDS data is regulated by French law and requires procurement of consent from the French data protection authority (CNIL). Any researcher from a European entity can submit an access request to the Health Data Hub (https://www.health-data-hub.fr). The study protocol can be provided to researchers upon a motivated request to the corresponding author.
