# Supplementary material for: Immunosuppressive treatment patterns in kidney transplant recipients in France: an insurance claims database study (OISTER) over a 12 year period
Source: J Nephrol. 2025 May 13;38(4):1229–37. doi: 10.1007/s40620-025-02296-4 (PMC12187887; doi:10.1007/s40620-025-02296-4)
Supplement: Supplementary file 1 — Supplementary file1 (DOCX 364 KB) [file 40620_2025_2296_MOESM1_ESM.docx]

## **SUPPLEMENTARY MATERIAL**

## **Supplementary Table 1. Codes used to retrieve morbidities from the database**

| **Variable** | |  |  |
| --- | --- | --- | --- |
| **Graft loss** | ≥1 of the following: |  |  |
|  | **EITHER** Transplantectomy | Procedure code | JADA015 |
|  | **OR** ≥38 haemodialysis sessions within a 90-day period | Procedure code  **OR** Disease group code  **OR** Service code | JVJF004, JVJF008, JVRP004  11K021, 11K022, 11K023, 11K024, 11K02J, 28Z03Z, 28Z04Z  2121, 2122, 2123, 2126, 2129, 2131, 2132, 2134, 2135, 2136, 2139, 2147, 2334 |
|  | **OR** ≥1 peritoneal dialysis session | Procedure code  **OR** Disease group code  **OR** Service code | JVJB001, JVRP007, JVRP008, YYYY007  28Z01Z, 28Z02Z  2124, 2125, 2127, 2128, 2137, 2138, 2140, 2142, 2143, 2144, 2145, 2146 |
|  | **OR** New renal transplantation | Procedure code | JAE003, HNEA002 |

| **Arteritis of the lower limbs** | ICD-10 code | I70.2, I73.9, I74.0, I74.3, I74.4 OR I74.5 |
| --- | --- | --- |
| **Ischemic heart disease** | ICD-10 code | I20, I21, I22, I23, I24 OR I25 |
| **Angina** | ICD-10 code | I20 |
| **Vascular stent** | Procedure code  **AND** | DDAF003, DDAF004, DDAF006, DDAF007, DDAF008 OR DDAF009 |
|  | Medical device code | 3155559, 3155559, 3142930, 8126611, 8115889, 8113146, 8112780, 8111160, 8130995, 3177696, 8126628, 3121187, 3166422, 3104190, 3191621, 3189340, 3180468, 3170754, 3190350, 3104496, 3177472, 3124955, 3121260, 3133931, 3147784, 3154761, 3145816, 3197730, 3150510, 3186666, 3113288, 3119581, 3165670, 3101010, 3146320, 3170926, 3152609, 3116186, 3110255, 3132104, 3127630, 3186778, 3173439, 3168036, 3133523, 3112811, 3143846, 3104941, 3165026, 3128841, 3170783, 3118475, 3165799, 3103976, 3108927, 3132593, 3126405, 3113360, 3126730, 3175148, 3102072, 3138822, 3115092, 3124352, 3125943, 3123536, 3160922, 3183604, 3159592, 3148677, 3121490, 3139649, 3113839, 3100185, 3196015, 3149671, 3148312, 3166310, 3143214, 3158144, 3182852, 3113934, 3132475, 3178632, 3112892, 3165635, 3164618, 3147790, 3134669, 3147867, 3168190, 3130192, 3133486, 3108293, 3181284, 3164529, 3111705, 3116424, 3146187, 3166764, 3153098, 3167700, 3126055, 3108695, 3176047, 3186809, 3179991, 3110999, 3118021, 3147809, 3134988, 3107081, 3119747, 3131961, 3199634, 3115643, 3123654, 3134712, 3150763, 3190165, 3118883, 3103864, 3150421, 3135960, 3192164, 3145294, 3164392, 3195843, 3139655, 3116507, 3168384, 3148588, 3164825, 3192856, 3138851, 3161519, 3104160, 3199574, 3196601, 3162973, 3196759, 3191986, 3181278, 3149978, 3160589, 3169188, 3158109, 3136177, 3196096, 3166014, 3138443, 3186844, 3164624, 3115175, 3180600, 3104881, 3152779, 3165173, 3171392, 3181210, 3183350, 3153135, 3135255, 3134379, 3171950, 3121388, 3125469, 3140894, 3133865, 3168289, 3194269, 3179465, 3136473, 3101411, 3176892, 3177740, 3165204, 3181226, 3152785, 3159304, 3129438, 3134735, 3136467, 3141356, 3107922, 3131010, 3157825, 3126782, 3133227, 3112834, 3102942, 3131174, 3137159, 3149085, 3102110, 3115933, 3164630, 3109051, 3149518, 3185827, 3112107, 3187619, 3174373, 3127050, 3115181, 3136450, 3121075, 3160626, 3122689, 3165641, 3102043, 3137194, 3190171, 3143869, 3158061, 3189251, 3125475, 3132860, 3186608, 3167611, 3113087, 3121980, 3129326, 3195553, 3112113, 3159563, 3161488, 3128700, 3109855, 3106207, 3136881, 3140701, 3122442, 3148393, 3107891, 3199752, 3167717, 3139550, 3154778, 3159178, 3109996, 3135396, 3113816, 3150160, 3114282, 3130542, 3154100, 3117872, 3123772, 3117040, 3161212, 3136651, 3102860, 3193169, 3107141, 3112219, 3186815, 3179985, 3159965, 3113437, 3116909, 3131978, 3131576, 3143906, 3153081, 3192796, 3161844, 3182289, 3185320, 3141853, 3159907, 3148401, 3153856, 3129728, 3163398, 3199580, 3180623, 3101138, 3164185, 3137308, 3115940, 3109571, 3187341, 3107997, 3178224, 3152650, 3110166, 3147034, 3171825, 3107856, 3168415 OR 3125587 |
| **Heart failure** | ICD-10 code | I50, I11.0, I13.0, I13.2 OR I13.9 |
| **Severe diabetes**  Diabetes with complications |  |  |
|  | ICD-10 code | G59.0, G63.2, G73.0, G99.0, H28.0, H36.0, I79.2, L97, M14.2, M14.6 OR N08.3 |
| Diabetes | ICD-10 code  **AND**  ATC Code | E10, E11, E12, E13 OR E14  At least two deliveries in pharmacies of ≥3 glucose-lowering drugs at the same date: A10A OR A10B (except for A10BX06) |
|  | **OR**  ATC Code | At least two deliveries in pharmacies of 2 glucose-lowering drugs at the same date: an insulin (A10A) **AND** any glucose-lowering drug (A10B except for A10BX06). |
| **Mild to moderate diabetes** | ICD-10 code  **AND**  ATC Code  **OR**  ATC Code + Packaging code | E10, E11, E12, E13 OR E14  At least three deliveries in pharmacies of any glucose-lowering drug: A10A, A10B except for A10BX06  Two deliveries in pharmacies of any glucose-lowering drug, of which at least one in a package containing >80 pills: A10A OR A10B except for A10BX06 |
| **Severe hypertension**  Hypertension with complications | ICD-10 code  **AND**  ATC Code | I11, I12, I13, I15 OR I67.4  At least two deliveries in pharmacies of ≥3 antihypertensive drugs at the same date: C02, C03, C07, C08, C09 OR C10BX03 |
| **Mild to moderate hypertension** | ICD-10 code  **AND**  ATC Code  **OR**  ATC Code + Packaging code | I10  At least three deliveries in pharmacies of any antihypertensive drug: C02, C03, C07, C08, C09 OR C10BX03 OR  Two deliveries in pharmacies of any antihypertensive drug, of which at least one in a package containing >80 pills: C02, C03, C07, C08, C09, C10BX03 |
| **Severe hyperlipidaemia**  Hyperliîdaemia with complications | ICD-10 code  **AND**  ATC Code | E78  At least two deliveries in pharmacies of ≥2 lipid-lowering drugs at the same date: C10 |
| **Mild-to-moderate hyperlipidemia** | ATC code  **OR**  ATC Code + Packaging code | At least three deliveries in pharmacies of any lipid-lowering drug: C10  Two deliveries in pharmacies of any lipid-lowering drug, of which at least one in a package containing >80 pills: C10 |
| **Chronic kidney disease** | ATC code  **OR**  Procedure code  **AND**  Service code  **AND**  Disease group code **OR**  Procedure code  **AND**  Service code  **AND**  Disease group code | N18  ≥19 hemodialysis sessions in inpatient or outpatient settings: JVJF004, JVJF008, JVRP004  2121, 2122, 2123, 2126, 2129, 2131, 2132, 2134, 2135, 2136, 2139, 2147, 2334  11K021, 11K022, 11K023, 11K024, 11K02J, 28Z03Z, 28Z04Z  ≥1 peritoneal dialysis session in inpatient or outpatient settings: JVJB001, JVRP007, JVRP008, YYYY007  2124, 2125, 2127, 2128, 2137, 2138, 2140, 2142, 2143, 2144, 2145, 2146  28Z01Z, 28Z02Z |
| **Stroke** | ICD-10 code | I60, I61, I62, I63, I64 |
| **Morbid obesity** | ICD-10 code  **AND**  Disease group code  **OR** Bariatric surgery  Procedure code | E66  10C091,10C092, 10C093,10C094, 10C101,10C102, 10C103, 10C104  HFCA001, HFCC003, HFKC001, HFKA002, HFFC018, HFFA011, HFMA010, HFMC006, HGCA009, HGCC027, HFMC007, HFMA009, HFGC900, HFLE002, HFFA001, HFFC004, HFKA001, HFLC900 |
| **Malnutrition** | ICD-10 code  **AND**  Disease group code | E43, E44, E46  10M181,10M182, 10M183,10M184, 10M18T,10M17T, 10M171,10M172, 10M173,10M174, 10M17T |

## **Supplementary Table 2. Treatment sequences over the follow-up period (2009-2019)**

|  | **INITIAL IMMUNOSUPPRESSIVE TREATMENT (N=29,709)^a^** | | | |
| --- | --- | --- | --- | --- |
|  | **Tacrolimus-based regimen** | **Ciclosporin-based regimen** | **mTOR inhibitors alone or in association** | **Belatacept-based regimen** |
|  | N=23,883 | N=4,678 | N=568 | N=137 |
| Continued throughout follow-up^b^ | 19,224 (80.5%) | 3,016 (64.5%) | 241 (42.4%) | 93 (67.9%) |
| Switched to ciclosporin | 1,330 (5.6%) | NA | 0 (0.0%) | <10 |
| Switched to tacrolimus | NA | 1,042 (22.3%) | 208 (36.6%) | 22 (16.1%) |
| Switched to mTORi alone or in association | 2,519 (10.5%) | 416 (8.9%) | NA | 17 (12.4%) |
| Switched to belatacept | 802 (3.4%) | 64 (1.4%) | 14 (2.5%) | NA |
| Switched to other IS drugs | <10 | 140 (3.0%) | 241 (42.4%) | 0 (0.0%) |
|  | **FIRST RELAY IMMUNOSUPPRESSIVE TREATMENT (N=6,760)^a^** | | | |
|  | **Tacrolimus-based regimen** | **Ciclosporin-based regimen** | **mTOR inhibitors alone or in association** | **Belatacept-based regimen** |
|  | N=1,389 | N=1,423 | N=3,111 | N=887 |
| Continued throughout follow-up^b^ | 1,169 (84.2%) | 1,078 (75.9%) | 1,649 (53.0%) | 524 (59.1%) |
| Switched to ciclosporin | 36 (3.1%) | NA | 120 (3.9%) | 12 (1.4%) |
| Switched to tacrolimus | NA | 167 (11.7%) | 950 (57.6%) | 285 (32.1%) |
| Switched to mTORi alone or in association in 3^rd^ line, | 42 (3.0%) | 66 (4.6%) | 222 (7.1%) (mTOR alone) | 42 (4.7%) |
| Switched to belatacept | 65 (0.4%) | 30 (2.1%) | 38 (0.4%) | NA |
| Switched to other IS drugs | 42 (3.0%) | 59 (4.1%) | 92 (3.0%) | <10 |

mTORI, mammalian target of rapamycin inhibitor. NA, not applicable.

^a^This total includes patients with other treatments, not presented in detail in the Table.

^b^Patients could have intercurrent interruptions of treatments. Interruptions of treatment were not considered as stopping, as long as it was the same treatment during the follow-up.

^c^The category of belatacept alone or in association includes the belatacept and mTOR inhibitor combination (this combination is minor (0.2% of the total of identified sequences (1^st^ to 4^th^ sequences), n=40,588).

## **Supplementary Figure 1. Number of transplantations per year**

The figures above or within the columns represent the number of transplantations in each year. Black columns: data from the present study (SNDS database); Yellow columns: data from the French national transplantation registry.

## **Supplementary Figure 2. Overall survival and graft survival as a function of age.**

| **A. Event-free survival (all patients)** |
| --- |
|  |
| **B. Graft survival (all patients)** |
|  |
| **C. Event-free survival (stratified by age)** |
|  |
| **D. Graft survival (stratified by age)** |
|  |

## **Supplementary Figure 3. Treatment patterns over time.**

| **** | **** |
| --- | --- |
| **** |  |

*NOTE*: No IS treatment was documented in 2,458 patients (7.6%)

The numbers in *italics* at the top of the graph represent the number of kidney transplant recipients in each calendar year. These numbers are used as the denominators for calculating the percentages. The numerators represent the number of patients grafted in a given calendar year who received the indicated treatment at least once at any time during follow-up.

CNI: calcineurin inhibitor; mTOR: mammalian target of rapamycin.
